# Colocolic intussusception secondary to colonic adenocarcinoma with impending caecal perforation in an elderly patient: A rare case report

**DOI:** 10.1016/j.ijscr.2022.107093

**Published:** 2022-04-19

**Authors:** Diptee Poudel, Shankar Raj Lamichhane, K.C. Ajay, Narendra Maharjan

**Affiliations:** a Maharajgunj Medical Campus, Institute of Medicine, Maharajgunj, Kathmandu, Nepal; b Department of Surgical Gastroenterology, Tribhuvan University Teaching Hospital, Maharajgunj, Kathmandu, Nepal

**Keywords:** Adult colocolic intussusception, Colon adenocarcinoma, Dilated caecum

## Abstract

**Introduction:**

Intussusception is a surgical emergency in which a part of the intestine slides into the distal adjacent part. Adult colocolic intussusception secondary to a tumoral process is a rare but serious clinical condition requiring immediate surgical intervention.

**Case presentation:**

We report a case of a 65-year-old male patient presenting with abdominal pain and distention, subsequently diagnosed with colocolic intussusception in the descending colon with closed-loop bowel obstruction with impending caecal perforation. An exophytic mass on the descending colon was discovered intra-operatively, prompting a subtotal colectomy with ileosigmoidal anastomosis and loop ileostomy with the suspicion of malignancy. The histopathological examination of the surgical specimen concluded a moderately-differentiated colonic adenocarcinoma with 40% mucinous component.

**Clinical discussion:**

Adult intestinal intussusception is a rare but serious condition differing greatly in etiology from its pediatric counterpart. Its preoperative diagnosis is challenging in adults, which appears to be due to its imprecise presenting signs and symptoms; thus, the condition can be mistaken for other causes of intestinal obstruction. Adenocarcinomas remain the most common cause of malignant tumors in the colon, which also makes them one of the causes for colocolic intussusception.

**Conclusion:**

Intussusception can appear as a surgical emergency even in the elderly, necessitating prompt surgical intervention to avoid intestinal ischemia and gangrene. Its diagnosis can be aided to a great degree by CT imaging.

## Highlights

• Colocolic intussusception is a rare surgical emergency in adults and is often associated with neoplastic lead points.
• Clinical diagnosis of the disease is challenging because of its non-specific presenting features. However, its diagnosis can be aided to a great degree by CT imaging.
• The disease necessitates prompt surgical intervention to avoid intestinal ischemia and gangrene.

## 1 Introduction

An intussusception is a form of bowel obstruction where there is telescoping of a bowel segment with its mesenteric fold (intussusceptum) into the adjacent distal bowel (intussuscipiens). Intestinal intussusception is infrequent in adults unlike pediatric intussusception, accounting for only around 5% of all intussusceptions cases [1]. In adults, intussusception constitutes only about 1% of all intestinal obstructions cases and is often secondary to a pathological condition while in children it is usually idiopathic [2], [3]. Intussusception can be classified based on its location (enteroenteric, ileocolic, or colocolic), etiology (idiopathic, benign, or malignant), and presence or absence of lead point. Among the three types of gastrointestinal intussusceptions classified based on their location, colocolic intussusceptions in the adults are the least prevalent type accounting for 8–19%, whereas, ileocaecal intussusceptions are the most common followed by enteroenteric intussusceptions.

In the majority of cases, the precipitating lesion is a bowel malignancy, thus making surgical resection the preferred choice of treatment for intussusception in adults [3], [4]. Other causes include post-trauma, Meckel's diverticulum, postoperative adhesions, lipomas, and adenomatous polyps [5].

In adults, intussusception presents with variable clinical manifestations. Since it rarely presents with the classic triad of vomiting, abdominal pain, and passage of blood per rectum, its preoperative clinical diagnosis is challenging. Abdominal Computed Tomography (CT) is the imaging modality of choice for the diagnosis of intussusception in adults. Pathognomonic signs of the condition include “target” or “sausage-shaped” masses in the outer intussuscipiens and inner intussusceptum [6].

In this study, we report a rare case of colocolic intussusception in an elderly patient. The patient was correctly diagnosed using an abdominal CT scan, which highlights the essential role of imaging in the diagnosis of intussusception in adults. The imaging also dictates the type of surgical intervention required for the patient. This case has been reported in line with the SCARE criteria [7].

## 2 Case presentation

A 65-year-old male presented to the emergency department with the chief complaint of insidious onset, colicky, moderately severe, and non-radiating abdominal pain for 1 week followed by 5 days of obstipation and a lump in the right side of the abdomen. He also provided a history of a non-bloody diarrheal episode about 2 weeks back. His appetite was reduced with an unintentional weight loss of 10 lbs. but he denied any history of fever, nausea, vomiting, hematochezia /melena. His family history was non-contributory and he had no significant past medical history. Social history of occasional alcohol consumption and tobacco use were positive. The patient denied any illicit drug use.

On examination, the patient was alert and his vital signs were normal. He was afebrile with a blood pressure of 110/80 mmHg, pulse rate of 80 beats per minute, respiratory rate of 18 breaths per minute, and SpO2 of 97% in room air.

On abdominal examination, mild tenderness was elicited on right lower quadrant without rebound tenderness and guarding. An ill-defined soft, smooth mass could be palpated on the right side of the abdomen with an extension from the right lumbar region to the right iliac fossa. The abdomen was distended with increased bowel sound without any sign of peritonitis. All the hernial sites were intact. On digital rectal examination, no mass could be felt but the glove was stained with blood. Other systemic examinations were found to be normal.

Laboratory investigations were within normal limits with hemoglobin of 12.3 g/dL [1], [2], [3], [4], [5], [6], [7], [8], [9], [10], [11], [12], [13], [14], [15], total leucocyte count of 10,000/μL, platelets of 359,000/μL, serum sodium of 146 mEq/L, serum potassium of 4 mEq/L, random blood sugar level of 5.1 mmol/L, urea of 3.3 mmol/L, and creatinine of 76 μmol/L.

**Fig. 1:**
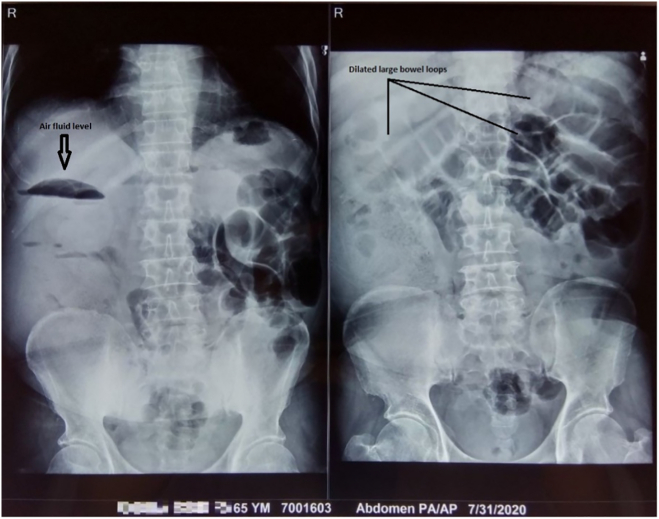
Erect abdominal X-ray showing dilated large bowel loops and air fluid level.

**Fig. 2:**
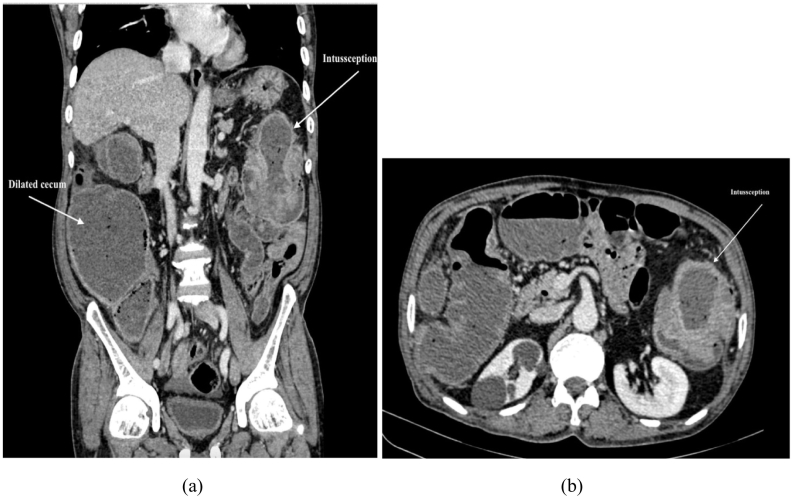
CT abdomen showing dilated caecum and telescoping of bowel segment into the descending colon.

An abdominal X-ray revealed multiple dilated large bowel loops with few air-fluid levels suggesting large bowel obstruction (Fig. 1). Ultrasonography of abdomen and pelvis demonstrated evidence of bowel within bowel appearance in the left upper quadrant of abdomen measuring 77 × 75 mm with target sign suggestive of intussusception of probably colocolic type along with fecal matter-containing dilated bowel loops in the right iliac fossa. Abdomino-pelvic CT scan showed left descending colon intussusception along with the classic target sign (Fig. 2).

**Fig. 3:**
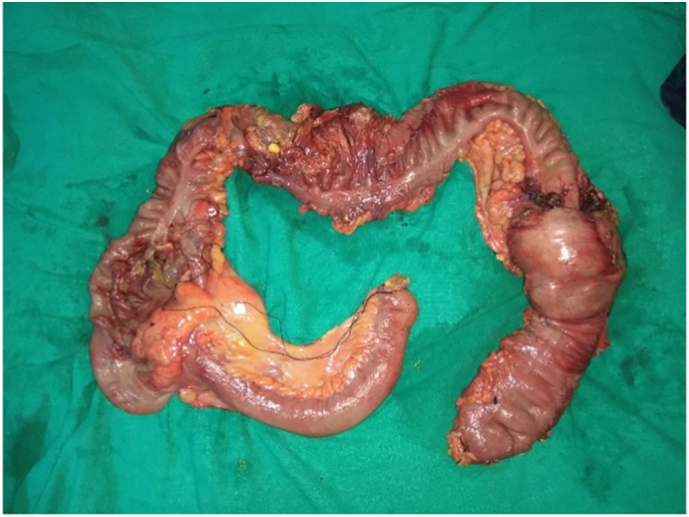
Subtotal colectomy specimen showing segment of ileum, ruptured caecum, appendix, ascending colon, transverse colon, and descending colon with colocolic intussusception.

The clinical and radiological findings in the patient established that this was a case of colocolic intussusception causing closed-loop intestinal obstruction due to a competent ileocecal valve. The patient was then promptly administered intravenous fluids and anitbiotics and underwent nasogastric decompression. Emergency laparotomy was done and on exploring the abdomen, a large bowel loop invaginating into the descending colon with a large colonic mass as the lead point was revealed. The proximal bowel loops were dilated with the caecum being hugely dilated with impending perforation. The colonic mass was suspected to be malignant in origin. Thus, a standard subtotal colectomy (Fig. 3) with ileosigmoidal anastomosis with loop ileostomy was performed without initial intussusception reduction. During the procedure, the massively dilated caecum burst out for which abdominal lavage with warm saline water was performed.

**Fig. 4:**
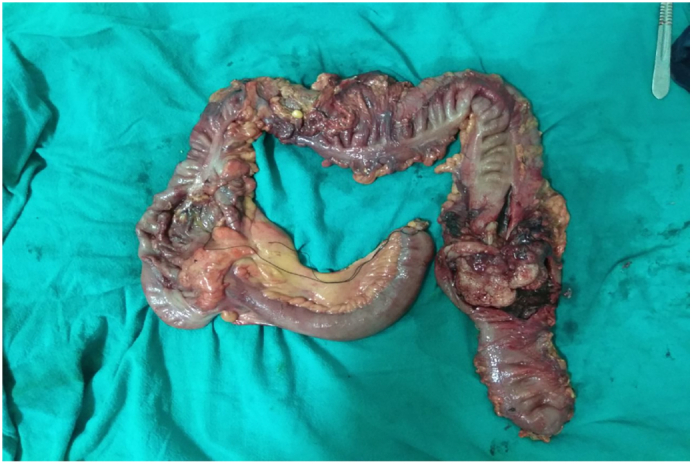
Cut section through the intussusception showing an exophytic friable mass in the descending colon, grey white to grey brown in color measuring 8.0 cm × 7.5 cm × 7.5 cm grossly involving up to muscularis propria. (For interpretation of the references to color in this figure legend, the reader is referred to the web version of this article.)

On opening the specimen, an exophytic, friable mass of size 8.5 cm × 7 cm × 7 cm was seen arising from the posteromedial wall of the descending colon (Fig. 4).

**Fig. 5:**
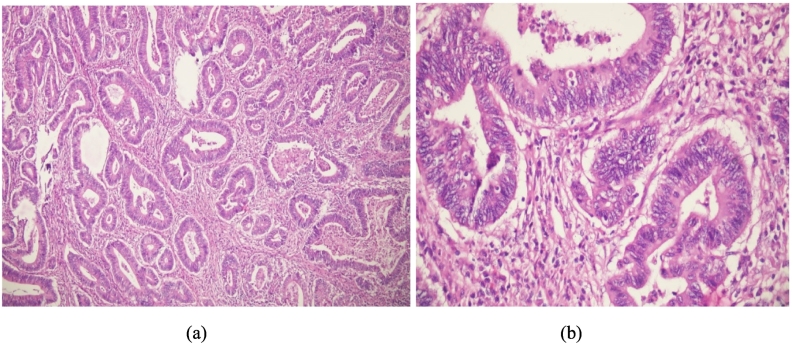
Section showing neoplastic tubular to cystic glands lined by pseudostratified columnar epithelium with loss of polarity and nuclear hyperchromasia. (a. H&E ×100, b. H&E ×400).

Histopathological examination of the cut sections from mass showed neoplastic tubular to cystic glands infiltrating the mucosal lamina propria, submucosa, muscularis propria, and perimuscular fibrous connective tissue. The glands were lined by pseudostratified columnar epithelium with loss of polarity and nuclear hyperchromasia. Mitosis constituted 5/10 HPFs and intraluminal necrosis was present. This led to the diagnosis of moderately differentiated adenocarcinoma with 40% of mucinous components (T3 N0 Mx) (Fig. 5).

The post-operative period was uneventful and the patient recovered well. On 6-month follow-up patient was doing well and disease free.

## 3 Discussion

Intussusception is an uncommon cause of intestinal obstruction in adults representing 1% of bowel obstructions and 0.003% to 0.02% of all hospital admissions [8]. It is an emergency condition requiring early diagnosis and management to prevent bowel gangrene and death. Any lesion in the bowel wall or irritant within the lumen that alters normal peristaltic activity is thought to be a potential precipitating factor for intussusception [9], [10]. Intussusception is mainly idiopathic in children, but it is frequently linked to a specific cause in adults. More than 80% of the cases of gastrointestinal intussusception are secondary to a malignant tumor, in particular, adenocarcinoma (a common cause of colorectal carcinomas), as seen in our patient [9], [11]. 65–70% of cases of large bowel intussusception are associated with malignant tumors, whereas small bowel intussusceptions are only associated with malignancy in 30–35% of cases [9].

An intussusception with a lead point can clinically present in a variety of ways. Most commonly patients experience intermittent or episodic pain, blood in stool, nausea, and vomiting. The complications such as bowel obstruction, infarction, hemorrhage, perforation, or peritonitis may change the clinical presentation. Neoplastic-related symptomatology may include weight loss, melena, constipation, generalized fatigue/malaise, and/or a palpable mass on physical examination [12]. There was complete bowel obstruction, obstipation, weight loss, and a palpable mass on physical examination which suggested the presence of a tumor.

Radiological investigations are extremely helpful for the diagnosis of intussusception. Abdominal X-ray shows air-fluid levels and dilated bowel loops related to the occlusive syndrome. Ultrasonography can be useful to visualize the classical target sign on a transverse view. Abdominal CT is the most sensitive study to diagnose intussusceptions with a diagnostic accuracy of 83% and is preferred to other imaging studies like colonoscopy, ultrasonography, X-ray, and small bowel series [13]. An abdominal CT scan confirms the diagnosis of the occlusive syndrome and shows the invagination, its precise location, and etiology. In CT, characteristic features include the target-like image or sausage-shaped soft tissue mass with a layering effect [13]. A similar target-like image is seen in the descending colon in this case.

Early surgical intervention is required soon after the diagnosis due to the high occurrence of bowel ischemia and bowel gangrene [5]. Most cases of adult intussusception are caused by bowel malignancies [3] which necessitates an adequate tumor resection.

In this case, dilated proximal bowel loops and a hugely dilated caecum with impending perforation was discovered on laparotomy. The excessive dilatation of the caecum may be due to a competent ileocecal valve in the presence of bowel obstruction. The excessive dilatation of the caecum may cause the appearance of fragile regions due to precarious vascular anastomosis as the caecum becomes dependent on parietal infusions. This increases the risk of perforation and hence resulting in fecal peritonitis, increasing morbidity and mortality [14].

In this case, an exophytic friable mass was found intraoperatively in the descending colon. Histopathological examination of the mass revealed moderately differentiated adenocarcinoma with 40% mucinous component. In practice, nearly 70% of colorectal adenocarcinomas are diagnosed as moderately differentiated carcinoma [15]. Attempts at reduction of the intussusception before resection should only be made if no pathological cause is present in the bowel. An older age is believed to be a contraindication to reduction given the greater likelihood of malignancy.

## 4 Conclusion

Intussusception of the colon in the elderly occurs rarely and in most cases is secondary to carcinoma. This article elaborates the case of an elderly man with colocolic intussusception due to adenocarcinoma of the descending colon. Thus, intussusception should be considered in the differential diagnosis of patients who present with a longstanding history of vague abdominal pain and weight loss. The workup must include additional radiographic studies like ultrasound or CT scan of the abdomen at the time of presentation. Finally, mandatory operative intervention is required after the diagnosis of intussusception in the elderly due to the high risk of malignancy.

## Sources of funding

None.

## Ethical approval

Not required.

## Consent

Written informed consent was obtained from the patient for publication of this case report and accompanying images. A copy of the written consent is available for review by the Editor-in-Chief of this journal on request.

## Author contribution

Diptee Poudel (DP) = Study concept, Data collection, Writing - original draft preparation.

Narendra Maharjan (NM), Shankar Raj Lamichhane (SRL), Ajay KC (AK) = surgical therapy for the patient.

Diptee Poudel (DP), Narendra Maharjan (NM), Shankar Raj Lamichhane (SRL), Ajay KC (AK) = Editing and writing.

All the authors read and approved the final manuscript.

## Registration of research studies

Not applicable.

## Guarantor

Dr. Diptee Poudel.

## Provenance and peer review

Not commissioned, externally peer-reviewed.

## Declaration of competing interest

The authors declare no competing interests.

## References

[1] Yalamarthi S., Smith R.C. (2005 Mar). Adult intussusception: case reports and review of literature. Postgrad. Med. J..

[2] Alshoabi S.A., Abdulaal O.M. (2019 Mar 15). An unusual case of colonic intussusception in old age. J. Taibah Univ. Med. Sci..

[3] Teyha P.S., Chandika A., Kotecha V.R. (2011 Aug). Prolapsed sigmoid intussusception per anus in an elderly man: a case report. J. Med. Case Rep..

[4] Yang C.K., Liang W.S., Liu C.K., Hsu H.H. (2015 Mar). Intussusception caused by colonic tumors in elderly patients: a case series of seven patients. Int. J. Gerontol..

[5] Marinis A., Yiallourou A., Samanides L., Dafnios N., Anastasopoulos G., Vassiliou I., Theodosopoulos T. (2009 Jan 28). Intussusception of the bowel in adults: a review. World J. Gastroenterol..

[6] Lu T., Chng Y.M. (2015 Winter). Adult intussusception. Perm. J..

[7] Agha R.A., Franchi T., Sohrabi C., Mathew G., Kerwan A., SCARE Group (2020 Dec). The SCARE 2020 guideline: updating consensus Surgical CAse REport (SCARE) guidelines. Int. J. Surg..

[8] Azar T., Berger D.L. (1997 Aug). Adult intussusception. Ann. Surg..

[9] Valentini V., Buquicchio G.L., Galluzzo M., Ianniello S., Di Grezia G., Ambrosio R., Trinci M., Miele V. (2016). Intussusception in adults: the role of MDCT in the identification of the site and cause of obstruction. Gastroenterol. Res. Pract..

[10] Wang N., Cui X.Y., Liu Y., Long J., Xu Y.H., Guo R.X., Guo K.J. (2009 Jul 14). Adult intussusception: a retrospective review of 41 cases. World J. Gastroenterol..

[11] Cavalleri A., Perrin H., Brunner P., Mourou M.Y., Bruneton J.N. (2007). Colocolic tumoral intussusception in the adult: value of multi-slice spiral CT imaging. Clin. Imaging.

[12] Boyack I., Vu D., Patel P., Opsha O. (2020 Aug). Colocolic intussusception secondary to submucosal lipoma. Am. J. Emerg. Med..

[13] Wilson A., Elias G., Dupiton R. (2013 Sep 5). Adult colocolic intussusception and literature review. Case Rep. Gastroenterol..

[14] Junior Toledo J.S., Correia M.M., Coutinho R.R., Kifer E.F., Torres D.F.M. (2017). Perforation of the cecum resulting from a closed-loop obstruction in a patient with an adenocarcinoma of the sigmoid colon: a case report. Int. J. Surg. Case Rep..

[15] Fleming M., Ravula S., Tatishchev S.F., Wang H.L. (2012 Sep). Colorectal carcinoma: pathologic aspects. J. Gastrointest. Oncol..

